# Supplementary material for: Agronomic or contentious land change? A longitudinal analysis from the Eastern Brazilian Amazon
Source: PLoS One. 2020 Jan 27;15(1):e0227378. doi: 10.1371/journal.pone.0227378 (PMC6984708; doi:10.1371/journal.pone.0227378)

**S1 Fig A.** **Deforestation trajectory on property that was excluded from our analyses after the balancing procedure.**

**
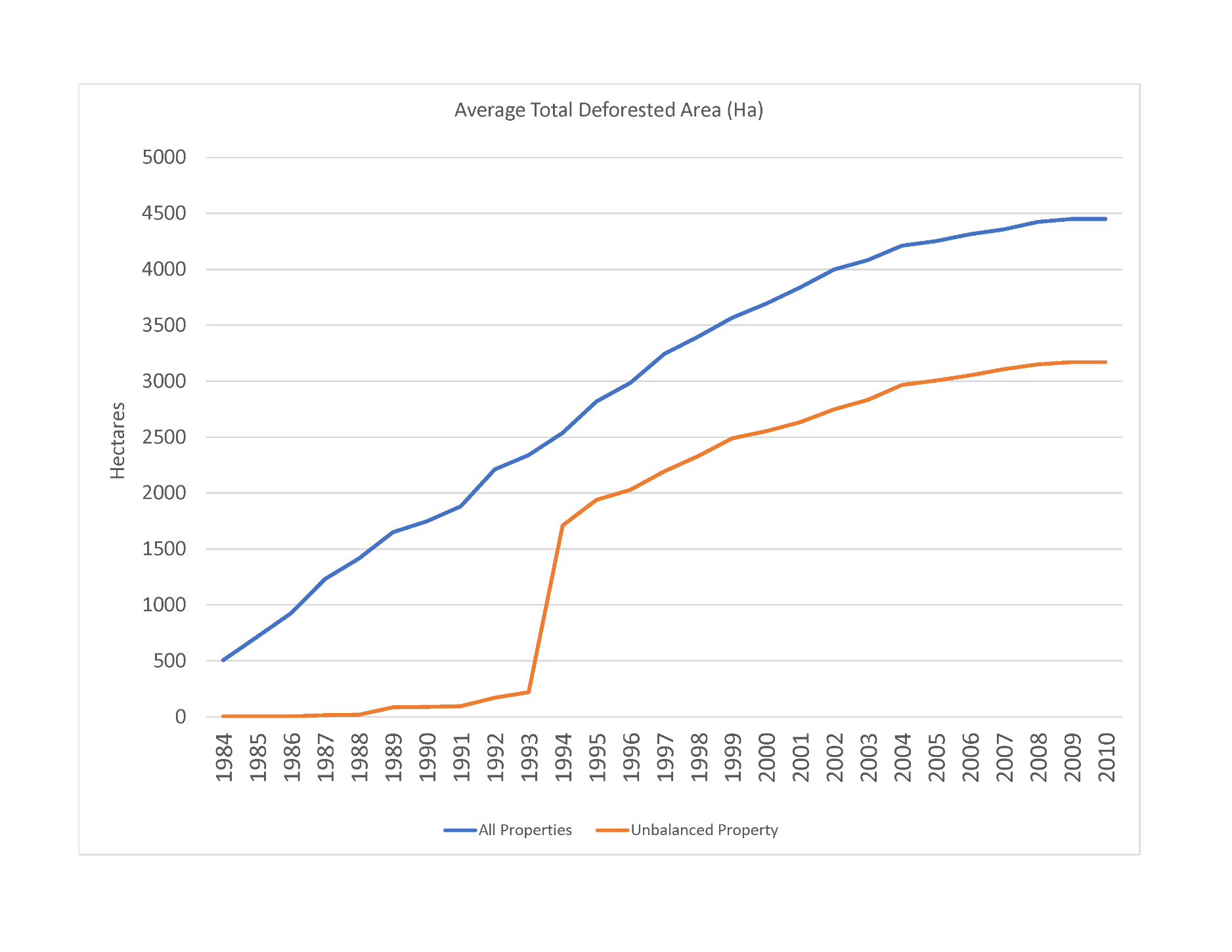
**

**S1 Fig B. Overlap Density Graph from Balancing Procedure**

The panel was well-balanced except for one property. That property experienced a relatively late, but very fast, uptick in deforestation. I have included in a graph comparing average deforested area on all properties versus the property that was excluded (labelled as "unbalanced property" in the SI). One of the assumptions required to use matching estimators successfully is the overlap assumption. This condition is satisfied when the probability of observing both occupied and non-occupied properties are similar given the combination of covariates. The graph shows substantial overlap in the mass density of both groups, **meaning that the overlap assumption is met**.


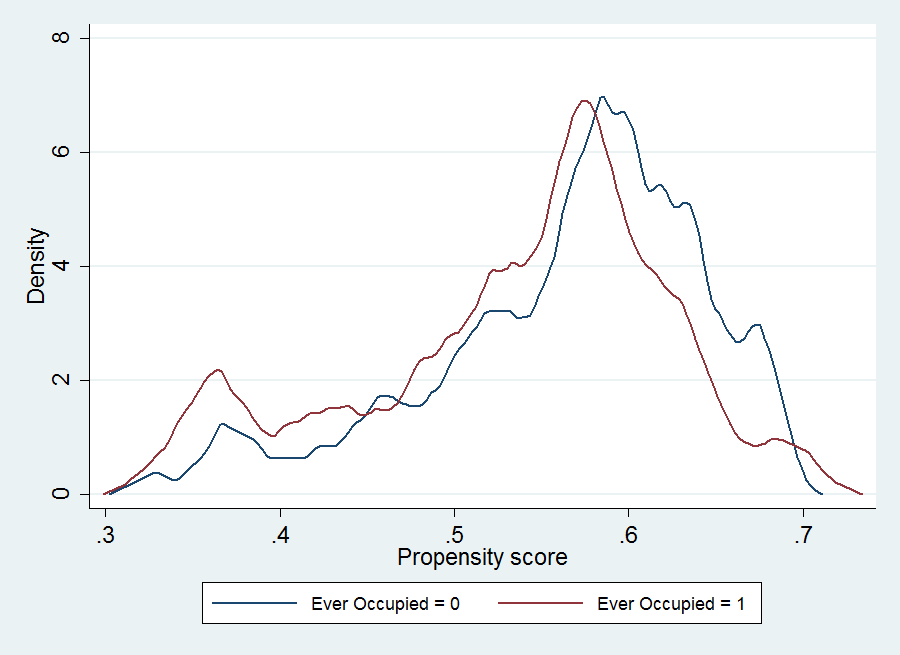

Supplement: S1 Fig — (DOCX) [file pone.0227378.s001.docx]
