# Supplementary material for: Agronomic or contentious land change? A longitudinal analysis from the Eastern Brazilian Amazon
Source: PLoS One. 2020 Jan 27;15(1):e0227378. doi: 10.1371/journal.pone.0227378 (PMC6984708; doi:10.1371/journal.pone.0227378)
Supplement: S1 File — (DOCX) [file pone.0227378.s002.docx]

**S1 File.** **Procedures for Newspaper Data**

The data on conflict events were accumulated from newspaper accounts collected from the two newspapers that were consistently published in the region during the study period (1984-2010). Both newspapers, *O Correio do Tocantins* (now known as "*O Correio do Carajás*") and *Opinão!* were published in Marabá, with a minimum of two issues per week (in the 1980s) up to daily publication (starting in 2001). These newspapers report exclusively in Portuguese, and one (*O Correio do Tocantins*) was subjectively more critical of land reform on its editorial pages than the other (*O Opinião!)*, which was more populist in its coverage of land conflict and land reform and its editorial pages were more likely to support landless social movements (although was still critical of their actions, overall).

The newspaper pages were photographed by author Aldrich at the *Casa da Cultura* (Cultural House), an archive and museum maintained by the Municipal government of Marabá in 2006, in 2010, 2011, and 2014. Photographs were taken using natural light (i.e., no flash, by an open window) at six megapixels using a Pentax K100D and a 50mm manual-focus lens, with supervision by the archivist on staff. Only pages with discussion of land tenure, land conflict, agrarian violence, or land reform-related events were photographed.

After the entire set of newspaper pages were photographed (a total of 8,572 photographs), each article was coded by author Aldrich (over 6,000 pages coded by Aldrich) or one of two Brazilian graduate assistants (approximately 2,572 coded by graduate assistants) under the supervision of author Castro. The coding process commenced with a complete reading of the article, start to finish, at which point the article would be assessed as being "codable" -- articles which did not includes specific information, such as op-eds, reports on policy discussions, or cases where specific events were mentioned to support an argument (rather than documenting those specific events) were considered codable. Codable articles were then manually read for keywords which frequently had quantitative data attached to them (the "salient details" we mention in the article). The Portuguese words for "police," "death," "attack," "expulsion," "eviction," "ranch/farm," "municipality," "gunmen," "injury," and "complaint," are all such keywords, though it should be stated that we did not develop a formal list of these keywords.

For each codable article, a new row in a standardized spreadsheet was created, and data on the event described was recorded. The headers of that spreadsheet are (in order): Date of Publication, Date of Event, Type of Event (organically developed list of descriptors, not used in analysis), Gunmen Present, Police Present, Number of Deaths, Ranch Name, Municipality, Largeholder Name, Description (a one-to-two sentence paraphrasing of the event), Newspaper Photo Filename.

Once the entire dataset of conflict details was complete, we cross referenced conflict events between both newspapers (based on date, location, and description of the events), and only those events described in both newspapers were included in the analysis presented in this paper.

Linking conflict events to location involved a manual hierarchical matching process. First, ranch names were compared (e.g., the newspaper might mention a ranch named "Cabaceiras") and we would review the ranches in the Brazil Nut Polygon that may have that name (as indicated by the cadastral map, described in the manuscript). If there was a match based on ranch name, we would then review whether that ranch was in the same Municipality as indicated in the newspaper article (e.g., newspaper indicated the ranch "Cabaceiras" is in the municipality of Marabá). If these two data points matched we would consider the conflict event to have taken place in the location indicated on the cadastral map. In the Brazil Nut Polygon there are twelve cases where different properties have the same name, but only two cases where those same names are located in the same municipality. In these cases we then compared largeholder names between newspaper events and the cadastral maps to refine the match. In the limited cases where this information was not available, we discarded the event from our analysis as it could not be geolocated appropriately.
