# Supplementary material for: Agronomic or contentious land change? A longitudinal analysis from the Eastern Brazilian Amazon
Source: PLoS One. 2020 Jan 27;15(1):e0227378. doi: 10.1371/journal.pone.0227378 (PMC6984708; doi:10.1371/journal.pone.0227378)
Supplement: S1 Table — (DOCX) [file pone.0227378.s003.docx]

**S1 Table.** **Variable Definition Table**

| **Variable Name** | **Definition** | **Range/Data Type** | **Source** |
| --- | --- | --- | --- |
| propids | Unique identifier for each property (unit of analysis) | 1-181 (nominal data) | SUDAM, "Perfil da ocupação do solo e subsolo Paraenses" (Profile of the occupation of the soil and subsurface claims of the State of Pará). Brasília, Brazil 1990. |
| year | Chronological year | 1984-2010 (ordinal) | N/A |
| propsize | Size of the property, measured in hectares. UTM Zone 22S, WGS1984 Datum. | 564-56035 (ratio data) | Geographic Information Systems calculation. |
| d2cities | Distance to urban centers, measured in kilometers | 3.89-79.3 (ratio data) | Cities locations provided by the Brazilian Institute of Geography and Statistics (IBGE), Euclidian distance calculated with Geographic Information Systems. |
| lhcontrol | Binary variable indicating periods of largeholder control. | 0-1 (nominal data) | Newspaper analysis (see SI-2) |
| numdalr | Number of conflict events (conflict of any kind). | 0-56 (ratio data) | Newspaper analysis (see SI-2) |
| numdeaths | Number of deaths recorded due to land conflict. | 0-24 (ratio data) | Newspaper analysis (see SI-2) |
| soilbin | Binary variable indicating if soils are suitable for smallholder agriculture (i.e., produce) | 0-1 (nominal data) | Soil map provided by Brazilian Agricultural Research Corporation (EMBRAPA), recoded using EMBRAPA soil descriptions. |
| def1whectares | Deforested area measured in hectares, no regrowth of forests allowed. | 0-55013 (ratio data) | Classified Landsat 5 scenes, land cover extracted to SUDAM map (see first row of this table). |
| settlementformed | Binary variable indicating if an agrarian reform settlement was formed. | 0-1 (nominal data) | National Agrarian Reform Institute (INCRA) agrarian reform settlement boundaries overlain on SUDAM map (see first row of this table) |
| dalrever | Binary variable indicating if property ever experienced conflict. | 0-1 (nominal data) | Recoding of numdalr variable. |
| balsamp | Binary variable indicating if observation should be excluded (exclude = 1) according to panel balancing (see methods discussion). | 0-1 (nominal data) | See methods section of main paper. |
| precipannual | Amount of precipitation per year. | 1347-2826 (ratio data) | NOAA/OAR/ESRL PSD, Boulder, Colorado, USA |
| period | Conflict epoch. | 1-3 (ordinal data) | Generated by authors based on history of conflict, Newspaper analysis (see SI-2). |
| run1 | Number of years since last land conflict event. | 0-27 (ratio data) | Newspaper analysis (see SI-2) |
| ddefha | First-difference deforested area (def1whectares). | 0-5481 (ratio data) | Derived from def1whectares variable. |
| dndalr | First-difference number of land conflicts (numdalr). | 0-56 (ratio data) | Derived from numdalr variable. |
| dndeaths | First-difference number of deaths (numdeaths). | 0-24 (ratio data) | Derived from numdeaths variable. |
| dlhcontrol | First-difference largeholder control (lhcontrol). | 0-1 (ratio data) | Derived from lhcontrol variable. |
| dprecip | First-difference precipitation (precipannual). | 0-1011 (ratio data) | Derived from precipannual variable. |
| dsettlementformed | First-difference settlement formed (settlement formed). | 0-1 (ratio data) | Derived from settlementformed variable. |
