# Supplementary material for: Agronomic or contentious land change? A longitudinal analysis from the Eastern Brazilian Amazon
Source: PLoS One. 2020 Jan 27;15(1):e0227378. doi: 10.1371/journal.pone.0227378 (PMC6984708; doi:10.1371/journal.pone.0227378)
Supplement: S2 Table — Note that lagging does not change the sign of the coefficient for conflict and agrarian reform settlement variables. (DOCX) [file pone.0227378.s004.docx]

**S2 Table. Lagged models for Hypothesis 1. Note that lagging does not change the sign of the coefficient for conflict and agrarian reform settlement variables.**

| **Dep. Variable:** | **Pooled OLS, First Difference Deforestation (Hectares), Detrended for Time** | **Fixed-Effects, First Difference Deforestation (Hectares)** | |
| --- | --- | --- | --- |
| **Regression Characteristics** | *n* = 4619, F [10, 4608] | *n* = 4619, F [7, 178] | |
|  | Prob > F = 0.0000 | Prob > F = 0.0000 | |
|  | R^2^ = 0.3276 | R^2^ = 0.0661 | |
| **Variable Name** | **Coefficient (t-value)** | **Coefficient (t-value)** | |
| **Number of Conflicts (Lagged)** | -0.639 (-0.26) | -41.800 (-1.76)* | |
| **Number of Deaths (Lagged)** | 3.054 (0.74) | 2.975 (0.83) | |
| **Settlement Formed (Lagged)** | 47.356 (6.79)*** | 69.785 (2.16)** | |
| **Years Since Last Conflict** | -0.831 (-1.95)* | -2.109 (-1.81)* | |
| **Annual Precipitation** | -0.056 (-4.82)*** | -0.051 (-5.20)*** | |
| **Property Size (Hectares)** | 0.029 (1.02) |  | |
| **Distance to Cities (Km)** | 0.217 (1.02) |  | |
| **Soil Binary** | -12.849 (-2.35)** |  | |
| **Time Period 1992-2001** | -44.167 (-4.39)*** | -41.257 (-3.05)** | |
| **Time Period 2001-2010** | -157.604 (-14.63)*** | -152.987 (-8.58)*** | |
| **Constant** | 170.799 (5.13)*** | 335.740 (14.83)*** | |
| Notes: Statistical significance indicated as follows: * = 0.10, ** = 0.05, *** = 0.000. | | |  |
