# Supplementary material for: Agronomic or contentious land change? A longitudinal analysis from the Eastern Brazilian Amazon
Source: PLoS One. 2020 Jan 27;15(1):e0227378. doi: 10.1371/journal.pone.0227378 (PMC6984708; doi:10.1371/journal.pone.0227378)
Supplement: S3 Table — Among properties with conflict those which are expropriated have significantly greater deforestation than those that were not. Note that 99 of the 180 properties had land conflict over the study period. (DOCX) [file pone.0227378.s005.docx]

**S3 Table. t-test on deforestation totals (1984-2010, measured in hectares) on contentious properties between those which were expropriated for settlement formation and those that were not. Among properties with conflict those which are expropriated have significantly greater deforestation than those that were not. Note that 99 of the 180 properties had land conflict over the study period.**

| **Group:** | ***N*** | **Mean** | **St.Err** |
| --- | --- | --- | --- |
| **Never Expropriated** | 43 | 3839.639 | 301.312 |
| **Expropriated** | 56 | 5669.159 | 986.013 |
| **Combined** | 99 | 4874.519 | 577.869 |
| **Difference** |  | -1829.521 | 1157.011 |
| **t** | -1.581 |  |  |
| H: Difference < 0  Pr(T<t) = 0.0585 | H: Difference <> 0  Pr(\|T\|>\|t\|) = 0.1171 | H: Difference > 0  Pr(T>t) = 0.9415 |  |
