# Supplementary material for: Agronomic or contentious land change? A longitudinal analysis from the Eastern Brazilian Amazon
Source: PLoS One. 2020 Jan 27;15(1):e0227378. doi: 10.1371/journal.pone.0227378 (PMC6984708; doi:10.1371/journal.pone.0227378)
Supplement: S4 Table — Related to this, deforestation and time are correlated, too. (DOCX) [file pone.0227378.s006.docx]

**S4 Table. Conflict and the passage of time are correlated (meaning that conflict is episodic, but concentrated in certain time periods). Related to this, deforestation and time are correlated, too.**

| **Dep. Variable:** | **Number of Conflict Events** | **Number of Conflict Events** | **Number of Conflict Events** |
| --- | --- | --- | --- |
| **Regression Characteristics** | *n* = 4860 | *n* = 4860 | *n* = 4860 |
|  | Prob > F = 0.0405 | Prob > F = 0.0406 | Prob > F = 0.0333 |
|  | R^2^ = 0.0007 | R^2^ = 0.0007 | R^2^ = 0.0.0010 |
| **Variable Name** | **Coefficient (t-value)** | **Coefficient (t-value)** | **Coefficient (t-value)** |
| **Year** | 0.001 (2.05)** |  |  |
| **Period**  **(1 = 1984-1992, 2=1992-2001, 3=2001-2010)** |  | 0.809 (2.05)** |  |
| **Period 2 (1992-2001)** |  |  | 0.191 (2.42)** |
| **Period 3 (2001-2010)** |  |  | 0.162 (2.05)** |
| **Constant** | -16.644 (-2.01)** | 0.127 (1.49) | 0.017 (3.06)** |
| Notes: Statistical significance indicated as follows: * = 0.10, ** = 0.05, *** = 0.000. | | | |
