# Supplementary material for: Agronomic or contentious land change? A longitudinal analysis from the Eastern Brazilian Amazon
Source: PLoS One. 2020 Jan 27;15(1):e0227378. doi: 10.1371/journal.pone.0227378 (PMC6984708; doi:10.1371/journal.pone.0227378)
Supplement: S5 Table — Properties without contention have significantly less deforestation than those that do, an outcome that supports H1. (DOCX) [file pone.0227378.s007.docx]

**S5 Table. t-test on deforestation totals (1984-2010, measured in hectares) between properties with conflict and those without. Properties without contention have significantly less deforestation than those that do, an outcome that supports H1.**

| **Group:** | ***N*** | **Mean** | **St.Err** |
| --- | --- | --- | --- |
| **No Contention** | 81 | 3503.346 | 227.902 |
| **Contention** | 99 | 4305.141 | 462.301 |
| **Combined** | 180 | 3944.333 | 275.119 |
| **Difference** |  | -801.795 | 551.294 |
| **t** | -1.454 |  |  |
| H: Difference < 0  Pr(T<t) = 0.0738 | H: Difference <> 0  Pr(\|T\|>\|t\|) = 0.1476 | H: Difference > 0  Pr(T>t) = 0.9262 |  |
