# Supplementary material for: Agronomic or contentious land change? A longitudinal analysis from the Eastern Brazilian Amazon
Source: PLoS One. 2020 Jan 27;15(1):e0227378. doi: 10.1371/journal.pone.0227378 (PMC6984708; doi:10.1371/journal.pone.0227378)
Supplement: S6 Table — (DOCX) [file pone.0227378.s008.docx]

**S6 Table. Arellano-Bond dynamic panel-data estimation for Table 2**

| **Dep. Variable:** | **Arellano-Bond dynamic panel-data estimation, First Difference Deforestation (Hectares)** | **Arellano-Bond test for zero autocorrelation in first-differenced errors** | | | **System dynamic panel-data estimation , First Difference Deforestation (Hectares)** |
| --- | --- | --- | --- | --- | --- |
| **Regression Characteristics** | *n* = 4261 | Order | Z | Prob > z | *n* = 4261 |
|  | Prob > chi2 = 0.0000 | 1 | -2.41 | 0.0161 | Prob > chi2 = 0.0000 |
|  |  | 2 | 1.17 | 0.2420 |  |
| **Variable Name** | **Coefficient (SE)** |  | | | **Coefficient (SE)** |
| **Lagged Deforestation** | 0.379 (0.04)*** |  | | | 0.399 (0.05)*** |
| **Number of Conflict Events** | 0.063 (1.44) |  | | | -0.608 (1.94) |
| **Number of Deaths** | -12.536 (5.41)** |  | | | -12.479 (5.57)** |
| **Settlement Formed** | 80.820 (89.41) |  | | | 133.823 (156.03) |
| **Years Since Last Conflict** | -3.465 (5.03) |  | | | -15.101 (3.19)*** |
| **Annual Precipitation** | -0.022 (0.009)** |  | | | -0.015 (0.02) |
| **Year** | -7.196 (2.82)** |  | | |  |
| **Constant** | 14518.18 (5601.84)** |  | | | 230.436 (48.53)*** |
| Notes: Statistical significance indicated as follows: * = 0.10, ** = 0.05, *** = 0.000. Robust Standard Error is presented. | | | | | |
