# Supplementary material for: Agronomic or contentious land change? A longitudinal analysis from the Eastern Brazilian Amazon
Source: PLoS One. 2020 Jan 27;15(1):e0227378. doi: 10.1371/journal.pone.0227378 (PMC6984708; doi:10.1371/journal.pone.0227378)
Supplement: S7 Table — (DOCX) [file pone.0227378.s009.docx]

**S7 Table. Arellano-Bond Dynamic Panel Data Estimation for Table 3b**

| **Dep. Variable:** | **Arellano-Bond dynamic panel-data estimation, First Difference Deforestation (Hectares) Detrended for Time, Contentious Properties Only** | **Arellano-Bond test for zero autocorrelation in first-differenced errors** | | | **System dynamic panel-data estimation , First Difference Deforestation (Hectares) Detrended for Time, Contentious Properties Only** |
| --- | --- | --- | --- | --- | --- |
| **Regression Characteristics** | *n* = 2338 | Order | Z | Prob > z | *n* = 2436 |
|  | Prob > chi2 = 0.0000 | 1 | -1.98 | 0.0476 | Prob > chi2 = 0.0000 |
|  |  | 2 | 1.02 | 0.3090 |  |
| **Variable Name** | **Coefficient (SE)** |  | | | **Coefficient (SE)** |
| **Lagged Deforestation** | 0.387 (0.04)*** |  | | | 0.432 (0.05)*** |
| **Number of Conflict Events** | 1.507 (1.67) |  | | | 3.371 (2.87 |
| **Number of Deaths** | -15.767 (5.41)** |  | | | -20.180 (7.56)** |
| **Largeholder Control** | -22218.53 (6417.38)** |  | | | 19964.97 (5545.88)*** |
| **Largeholder Control * Year** | 11.038 (3.19)** |  | | | -10.103 (2.80)*** |
| **Settlement Formed** | 112.524 (110.62) |  | | |  |
| **Years Since Last Conflict** | -0.996 (3.64) |  | | | 103.498 (91.92) |
| **Annual Precipitation** | 0.021 (0.014) |  | | | -7.869 (4.27)* |
| **Year** | -20.118 (4.00)*** |  | | | -0.040 (0.02)* |
| **Constant** | 40433.89 (8016.99)*** |  | | | 338.876 (97.66)** |
| Notes: Statistical significance indicated as follows: * = 0.10, ** = 0.05, *** = 0.000. Robust Standard Error is presented. | | | | | |
