# Supplementary material for: Agronomic or contentious land change? A longitudinal analysis from the Eastern Brazilian Amazon
Source: PLoS One. 2020 Jan 27;15(1):e0227378. doi: 10.1371/journal.pone.0227378 (PMC6984708; doi:10.1371/journal.pone.0227378)
Supplement: S8 Table — (DOCX) [file pone.0227378.s010.docx]

**S8 Table. Arellano-Bond test for Table 3c**

| **Arellano-Bond test for zero autocorrelation in first-differenced errors** | | |
| --- | --- | --- |
| Order | Z | Prob > z |
| 1 | -1.98 | 0.0481 |
| 2 | 0.099 | 0.3232 |
