# Supplementary material for: Agronomic or contentious land change? A longitudinal analysis from the Eastern Brazilian Amazon
Source: PLoS One. 2020 Jan 27;15(1):e0227378. doi: 10.1371/journal.pone.0227378 (PMC6984708; doi:10.1371/journal.pone.0227378)
Supplement: Attachment — Submitted filename: Response to Reviewers 20191112.pdf [file pone.0227378.s012.pdf]

Indiana State University
More. From day one.
Department of Earth & Environmental Systems
Terre Haute, Indiana 47809
812-237-2444
Fax 812-237-8029

November 12, 2019

Dear Dr. Nóbrega, Dr. Klinger (Reviewer 1), and Reviewer 2:

Thank you very much for your thoughtful advice and recommendations regarding our manuscript titled "Agronomic or Contentious Land Change? A Longitudinal Analysis from the Eastern Brazilian Amazon" which we submitted to PLOS ONE earlier this year. We appreciate your time and effort in helping us improve this manuscript, and have reviewed these comments and suggestions. They led us to make changes to the manuscript as detailed below.

- Regarding access to the data underlying the findings in this manuscript: the complete data necessary to reproduce these findings were included in the supporting information for the manuscript. It appears that both reviewers may have missed this, so we have also deposited the dataset necessary to reproduce these results in the Institutional Data Repository at Indiana State University, called Sycamore Scholars, available here: http://scholars.indstate.edu/

- Reviewer 1 suggestions:

    - More information regarding newspaper data: Thank you for pointing out the lack of detail in this important section. We have added text to section 3.b.ii (titled "Data") regarding how newspapers were selected (they were the two newspapers reliably published) and how the events we uncovered were linked to properties. We have also added a new section in the Supporting Information (SI-2) regarding the format and coding process for extracting information from newspaper articles (they were photographs of paper copies, taken by the first-author, numbering over 8,000 pages).

    - Figures and Tables: The caption on Figure 1 has been extended and Figure 1 has been revised to reflect the very useful suggestions. We also changed the attribute mapped to more accurately reflect land cover before the time period of analysis. Thank you! Figures 3 and 4 now have a labelled y-axis (apologies). The correct Figure 5 is now included. Typo on Page 29 is corrected (thank you!).

    - Language and Writing Style: We have removed or revised the words mentioned, although point out that the loss of more than 100 lives in this area due to land conflict is indeed horrific and unfortunate. We suggest that an article on objectively tragic events should not diminish their tragedy, but take your point on the style. The word "almost" has been largely expunged (a very valuable catch, thank you), and where it remains a value is explicitly associated with it (generally in parenthetical).

    - References: We greatly appreciate the suggestions for additional literature. We have incorporated these. We have also updated current report references where appropriate

(One CPT reference has been updated, DataLuta has not been published for 2018 as of October 22, 2019).

- Reviewer 2 Suggestions:

  - Variable definitions: We added a table including variable information in the supplemental information (SI-1). The data itself were attached to the first version of the manuscript, and there is a codebook in the same compressed archive as the data itself.

  - Conflict Endogeneity and Deforestation History: Because the comments on the potential endogeneity of conflict and comments about deforestation history as an explanatory variable are somewhat similar, we will address both comments here. Reviewer 2 raises the prospect of potential endogeneity of the variable 'conflict.' Such endogeneity can arise from two sources. First, conflict is endogenous if the estimated model omits a variable that is correlated with the independent variables; therefore the error term (now in the presence of the omitted variables) will be correlated with 'conflict' causing OLS procedures to be inconsistent (so called omitted-variable bias). Second, conflict endogeneity could arise from a reverse causality problem; here if deforestation causes conflict. In a cross-sectional setting (i.e. no time dimension), the inclusion of a lagged dependent variable (here deforestation in a previous period) would solve the endogeneity problem because a) previous deforestation would be a good instrument for omitted variables, and b) previous deforestation enters as an explanatory variable for conflict. This would also address the comment on 'deforestation history' for the same reasons as described in (b). Originally, we did not include lagged deforestation for a few reasons: a) we believe that we had included a rich set of explanatory variables that are known in the literature to be associated with deforestation (e.g. distance variables, soil quality, precipitation, among others). Therefore, I believed the 'omitted variable bias' problem was not an issue; b) in our methods, we adopted a matching estimator procedure prior to running the panel regressions. In the matching component, we included deforestation as an explanatory variable for conflict. Therefore, the properties used in the panel regressions are balanced: there is no bias with respect to conflict occurring in properties that have more (or less) forests; c) the inclusion of a lagged dependent variable would obviously increase the explanatory power of the model but would "suck out" the effect of the other variables, and therefore should only be used if there is a legitimate theoretical reason for it; d) In a *panel* framework (as opposed to a cross-sectional setting), the inclusion of lagged dependent variables in the model will, *by definition*, necessarily be endogenous. Having said that, we do see merits in the reviewer's suggestion about including a lagged dependent variable. Therefore, we show the results of two models: the (biased) pooled OLS that includes a lagged dependent variable, and an Arellano-Bond (AB) panel estimator that deals with endogeneity problems once the lagged dependent variable is included in panel estimators. We ran a third model (Arellano-Bover-Blundell-Bond) but do not report the results because they are very similar to the AB model. In general, the models are consistent in showing the effect of settlements on deforestation, and of violent conflicts (number of deaths).

- Title Status: We do have information on *alleged* title status. However, title status does not matter for our fixed effects models since it is time-invariant (at least over the time period of our analysis -- the endpoint of our analysis is 2010, when the properties were all formally privatized by state decree).

- Conceptual Model Section and 50% Forest Mandate: Our conceptual model, presented graphically in Figure 2 and described on page 12 should not be considered an empirical model. In fact, the empirics (shown in our models throughout the paper) show that this law is ignored by landholders irrespective of conflict. The results led us to revise this conceptual model, which is shown in Figure 5 (which was inadvertently not included in the submission of the first manuscript). If we look at the legal reserve requirement we find that 100% of the properties do not adhere to those requirements (i.e., none of the properties remain >49% forest cover in 2010). We did test this as suggested, using the interaction of stock forest (forest cover in 1984, the start of our analytical period) and number of conflict events, and in a first difference fixed-effects specification that interaction is not significant and the sign, magnitude, and significance of our other independent variables are not substantially different.

- More Description of Table 1: Thank you for this suggestion. We have added text reflecting our expectations to pages 18 and 19. Our conceptualizations of the relationship between these dependent variables are in-line with the broader land change literature.

- Roads and Measurement Error: There are no better roads data available. However, if we run our models using Euclidian distance to cities only the results are not substantially different. There is no adequate way to address this issue, but it is a well-known problem with meso-to-broad-scale analyses in the Brazilian Amazon.

- More Information on Panel Balancing: The panel was well-balanced except for one property. That property experienced a relatively late, but very fast, uptick in deforestation. I have included a graph comparing average deforested area on all properties versus the property that was excluded (labelled as "unbalanced property" in the SI, See SI-2A and SI-2B). One of the assumptions required to use matching estimators successfully is the overlap assumption. This condition is satisfied when the probability of observing both occupied and non-occupied properties are similar given the combination of covariates. The graph shows substantial overlap in the mass density of both groups, meaning that the overlap assumption is met.

- On Issues of Spatial Autocorrelation in Fixed Effect Models: We appreciate Reviewer 2's concern about "spatial autocorrelation" and set to clarify our initial citation. If the concern is about spatial autocorrelation of the error term, robust estimators used here should attenuate the problem, which is what we meant by the citation provided in the text. Although the reviewer uses the term 'autocorrelation,' the description provided seems to indicate an issue with a spatial autoregressive process, the spatial analog of the inclusion of a lagged dependent variable in the time series framework. According to the reviewer

"deforestation in one property tends to correlate with deforestation in neighboring properties." While this statement is true, we disagree that this is the correct motivation for implementing a spatial autoregressive model (SAR, according to LeSage and Pace's 2009 terminology). What would be a data generating process that would justify the SAR model? We can think of two, none of which are present in the study area: first, a rancher would deforest their properties because their neighbors are doing it, not because of economic motivation but simply because he or she copies the behavior of the neighbor, which is unlikely. If motivation is economic, then it is already controlled for in our models (e.g. soils, distance to roads, precipitation). Second, if social movements occupy one property and decide to occupy another property simply because it is neighbor of the first. Again, our variable conflict would provide the necessary control here. SAR models greatly improve the explanatory power of the overall model (i.e. higher R-squares) but it come at a cost of ignoring causality and the ability to identify the effect of variables of interest. Partly this is because spatial econometrics was derived from time-series econometrics where prediction is center stage, not causality. For a good discussion of these issues, please refer to Gibbons and Overman (2012). Lastly, we accepted the reviewer's suggestion and added a lagged dependent variable as explained above. A model with both spatial and temporal lagged dependent variables would be hard to justify theoretically in the context of partial effects measurement. We can imagine a data generating process where one rancher deforests more because the neighbor's property was occupied. This data generating process is incorporated through an SLX model (spatial lag of X – conflict) and is a legitimate empirical question. In fact, we are in the process of examining this hypothesis for another paper. The hypotheses we seek to test in the current manuscript are different.

We appreciate the opportunity to revise our manuscript and hope that our efforts to address the very helpful comments by both reviewers are satisfactory.

Sincerely,

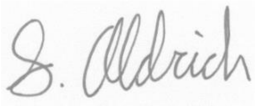

Steve Aldrich, PhD
Associate Professor of Geography
Department of Earth and Environmental Systems

References:

LeSage, J., & Pace, R. K. (2009). *Introduction to spatial econometrics*. Chapman and Hall/CRC.

Gibbons, S., & Overman, H. G. (2012). Mostly pointless spatial econometrics?. *Journal of Regional Science*, *52*(2), 172-191.
